# Annotating neurophysiologic data at scale with optimized human input

**DOI:** 10.1088/1741-2552/ade402

**Published:** 2025-07-03

**Authors:** Zhongchuan Xu, Brittany H Scheid, Erin C Conrad, Kathryn A Davis, Taneeta Ganguly, Michael A Gelfand, James J Gugger, Xiangyu Jiang, Joshua J LaRocque, William K S Ojemann, Saurabh R Sinha, Genna J Waldman, Joost Wagenaar, Nishant Sinha, Brian Litt

**Affiliations:** 1 Department of Bioengineering, School of Engineering and Applied Sciences, University of Pennsylvania, Philadelphia, PA 19104, United States of America; 2 Center for Neuroengineering and Therapeutics, University of Pennsylvania, Philadelphia, PA 19104, United States of America; 3 Department of Neurology, Perelman School of Medicine, University of Pennsylvania, Philadelphia, PA 19104, United States of America; 4 Department of Biostatistics, Epidemiology & Informatics, Perelman School of Medicine, University of Pennsylvania, Philadelphia, PA 19104, United States of America

**Keywords:** human-in-the-loop, seizure detection, epilepsy, active learning, self supervised learning, iEEG, annotation

## Abstract

*Objective.* Neuroscience experiments and devices are generating unprecedented volumes of data, but analyzing and validating them presents practical challenges, particularly in annotation. While expert annotation remains the gold standard, it is time consuming to obtain and often poorly reproducible. Although automated annotation approaches exist, they rely on labeled data first to train machine learning algorithms, which limits their scalability. A semi-automated annotation approach that integrates human expertise while optimizing efficiency at scale is critically needed. To address this, we present Annotation Co-pilot, a human-in-the-loop solution that leverages deep active learning (AL) and self-supervised learning (SSL) to improve intracranial EEG (iEEG) annotation, significantly reducing the amount of human annotations. *Approach.* We automatically annotated iEEG recordings from 28 humans and 4 dogs with epilepsy implanted with two neurodevices that telemetered data to the cloud for analysis. We processed 1500 h of unlabeled iEEG recordings to train a deep neural network using a SSL method Swapping Assignments between View to generate robust, dataset-specific feature embeddings for the purpose of seizure detection. AL was used to select only the most informative data epochs for expert review. We benchmarked this strategy against standard methods. *Main result.* Over 80 000 iEEG clips, totaling 1176 h of recordings were analyzed. The algorithm matched the best published seizure detectors on two datasets (NeuroVista and NeuroPace responsive neurostimulation) but required, on average, only 1/6 of the human annotations to achieve similar accuracy (area under the ROC curve of 0.9628 ± 0.015) and demonstrated better consistency than human annotators (Cohen’s Kappa of 0.95 ± 0.04). *Significance*. ‘Annotation Co-pilot’ demonstrated expert-level performance, robustness, and generalizability across two disparate iEEG datasets while reducing annotation time by an average of 83%. This method holds great promise for accelerating basic and translational research in electrophysiology, and potentially accelerating the pathway to clinical translation for AI-based algorithms and devices.

## 1. Introduction

Epilepsy is at the forefront of neurotechnology innovation, with devices that continuously monitor and manage neurological conditions producing rich longitudinal data that advance diagnosis and treatment [1–5]. However, this data surge has outpaced our ability to analyze it efficiently: analyzing these large datasets still depends heavily on expert human annotation, a process that is time-consuming, labor-intensive, and often inconsistent [6–8]. Yet these annotations remain essential for regulatory approval, clinical interpretation, and research analysis [9]. However, existing automated annotation algorithms, commonly tailored to specific devices or datasets, struggle to generalize without extensive adjustments. As a result, new models often still need substantial manual labeling for training and refinement [10]. This challenge underscores the need for a semi-automated annotation approach that incorporates human expertise more efficiently, improving scalability of analyses and ultimately enhancing clinical utility of these neurotechnologies.

Supervised and unsupervised machine learning have both been applied to address the challenge of annotating intracranial EEG (iEEG) data for various tasks such as seizure detection [11–16]. Supervised learning methods require extensive labeled datasets, where each data segment is manually annotated to train algorithms that can recognize seizure patterns. While these methods can achieve high accuracy, the labor-intensive nature of generating labeled data limits their scalability and generalizability, especially across heterogeneous patient populations and device configurations [17]. Unsupervised learning, on the other hand, clusters iEEG data without labels to identify patterns and similarities across clips. Although promising for rapid, large-scale data analysis, unsupervised approaches lack the precision needed for clinical applications, as they do not inherently distinguish seizure from non-seizure events [18].

This gap highlights the potential of human-in-the-loop (HITL) semi-supervised learning, where initial automated clustering is combined with expert oversight to refine labels iteratively. Unlike fully supervised methods, which rely on extensive labeled datasets that are resource-intensive to produce, HITL optimizes human input by directing expert attention to ambiguous or high-value data points, providing insights for complex or edge cases [9, 19, 20]. The process continues to update the model until new information no longer contributes to further improving the accuracy. By leveraging minimal labeled data and integrating expert intervention only where necessary, HITL approaches achieve a balance between efficiency and accuracy [21]. This approach bridges the gap between algorithmic efficiency and the nuanced judgment of human experts, making it a promising strategy for advancing both research and clinical applications.

In this work, we introduce Annotation Co-pilot, a HITL active learning (AL) approach designed to efficiently annotate iEEG data, reducing the reliance on exhaustive manual labeling while maintaining high accuracy and consistency in seizure detection. As a proof of concept, we applied our method to data from the NeuroPace responsive neurostimulation (RNS) [22] and NeuroVista Seizure Advisory System [23]. Our method combines self-supervised learning (SSL) [24–26] to extract meaningful features from large volumes of unlabeled data with deep AL [27, 28] to strategically select the most informative samples for annotation. This dual strategy minimizes the annotation burden on experts while enabling the model to learn from a fraction of the labeled data typically required by traditional supervised approaches.

## 2. Methods

### 2.1. Descriptions of the datasets

**RNS dataset** The RNS Dataset consists of 60 876 clips of intracranial recordings collected as a part of routine clinical care from the RNS system across 28 patients at the Hospital of the University of Pennsylvania (HUP) [22]. The RNS device is an implantable neurostimulator that monitors neural activity and delivers electrical stimulation when abnormal EEG is detected. The device records short clips, approximately 90 s in duration, during two daily scheduled times (scheduled event clips) and during prolonged abnormal activity (long episode clips). Each recording includes four channels sampled at 250 Hz, and is discontinuous between episodes. RNS data collection for research was approved by the HUP institutional review board and informed consent was obtained from each subject. The cohort comprised 28 patients—18 women and 10 men. Seizure onset ranged from 1 to 46 years of age, and RNS devices were implanted between ages 18 and 76. Electrode leads were unilateral in 12 patients and bilateral in 16, targeting neocortical, mesial-temporal, or combined mesial/neocortical foci across the frontal, temporal, and parietal lobes. Recordings from a subset of patients were annotated by expert clinicians. Examples of this dataset are provided in appendix G.

**NeuroVista Dataset** The NeuroVista dataset, containing 23 844 1 s recordings of canines with naturally occurring epilepsy, was collected using the NeuroVista Seizure Advisory System [23], an implantable device. iEEG recordings were obtained from 16 subdural electrodes on two 4-contact strips implanted bilaterally in an antero-posterior configuration, sampled at 400 Hz with an anti-aliasing low-pass filter (100 Hz and 150 Hz poles). These segments were derived from longer annotated recordings and then shuffled for use in a Kaggle competition [29]. This dataset is publicly available, and will be used as an external benchmark for testing the generalizability of the pipeline. Examples of this dataset are provided in appendix A.

### 2.2. Annotation acquisition and inter-rater reliability

**Figure 1.**
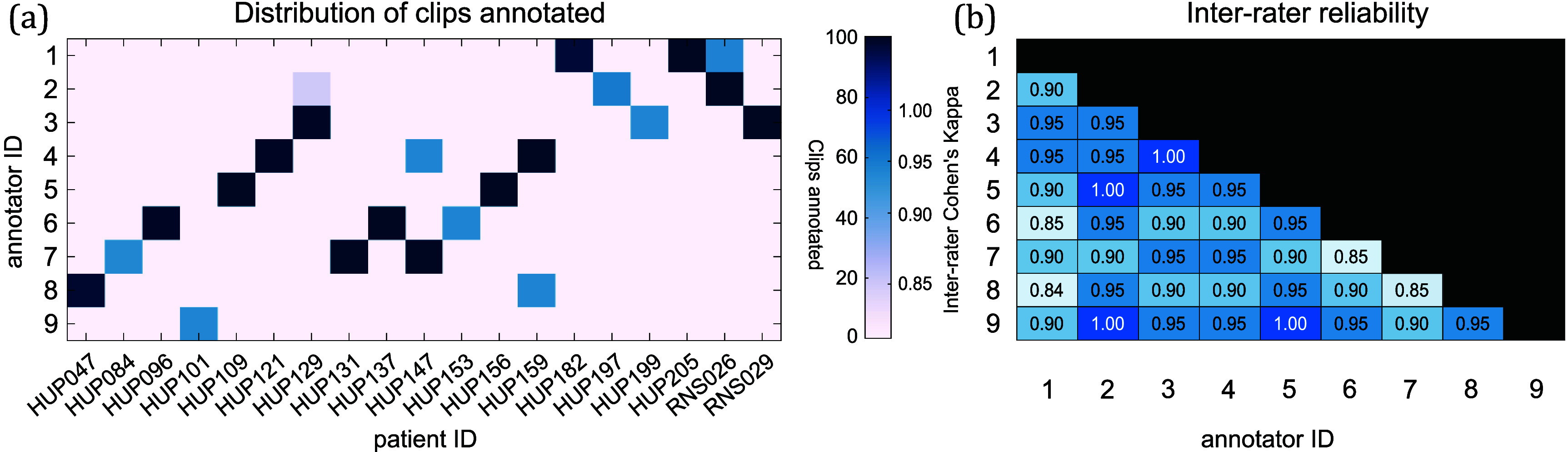
Data annotation seizure vs non-seizure clips (a) the number of clips (color shading in squares) annotated as seizure or not seizure for each subject (*x*-axis) by each of 10 expert reviewers (*y*-axis left). Scale for shading is labeled on *y*-axis right on a scale of 0–100. (b) inter-rater agreement between marking experts on 50 segments annotated by all experts shows percentage agreement for each pair of experts.

**RNS dataset annotations** Nine board-certified epileptologists (BL, EC, GW, JG, KD, TG, MG, SS, JL) annotated RNS data clips from 16 patients to establish ground truth labels, which were directly used to train and evaluate the algorithm. To assess inter-rater reliability, all clinicians first annotated the same test set of 50 clips randomly selected from three patients, maintaining a 7:3 ratio of long episodes to scheduled events. Additionally, each epileptologist annotated 250 clips randomly selected from three patients, with 50 of those clips also reviewed by a second annotator to assess interrater reliability (see figure 1).

Annotations were performed using Pennsieve, an open-source web platform for EEG data annotation [30]. Clinicians marked whether each clip included a seizure (‘yes,’ ‘no,’ or ‘maybe’) and, if applicable, annotated the unequivocal earliest onset and seizure offset [31]. Seizures beginning or ending at the clip’s edge were marked accordingly. Episodes where seizures were interrupted by stimulation but continued were also labeled as seizures. A total of 1400 annotated clips were ultimately collected with an overall ictal to interictal recording ratio of 1:4. table 2 summarizes patient information for those with any annotated recordings.

**NeuroVista dataset annotations** Recordings were reviewed, and seizures were annotated by two board-certified epileptologists (GW and BL). Non-functional or grossly non-physiological iEEG channels were excluded through visual inspection. The continuous recordings were annotated with seizure onset, end of early seizure, and seizure offset markers. These annotated recordings were then segmented into 1 s windows [29]. Segments between seizure onset and the end of the early seizure phase were labeled ‘early onset’; those between the end of the early onset phase and seizure offset were labeled ‘ictal’; and all remaining segments were labeled ‘interictal.’ The ratios of early onset, ictal, and interictal segments were 1:2:26.

**RNS dataset annotation inter-rater reliability** We calculated inter-rater reliability using Cohen’s Kappa (*κ*) [32], which accounts for chance agreement. Among the 50 overlapping segments annotated by all experts, the results showed substantial agreement across annotators (figure 1). The reliability was assessed based on whether or not each clip was annotated as containing a seizure. Among the 50 overlapping segments annotated by all experts, *κ* values were calculated to measure consistency. The performance of the machine learning model will be compared against these human annotations to evaluate its accuracy and reliability.

### 2.3. Model design and data processing

**Figure 2.**
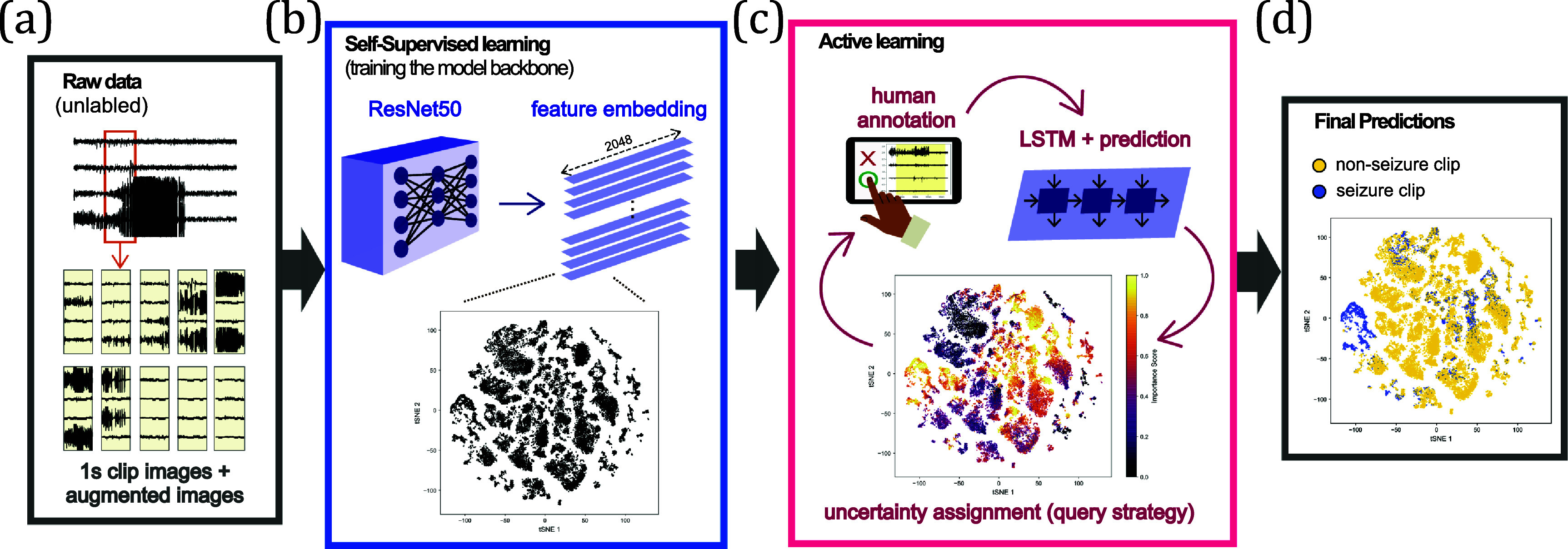
Pipeline overview. The proposed pipeline for iEEG data classification has several stages. (a) Initially, the unlabeled data is segmented into 1 s sliding window, with voltage values converted into grayscale pixels. Following data augmentation, (b) these clips are used to train a ResNet-50 model using self-supervised learning (SwAV) without labels. (c) Active learning is then employed to train an long short-term memory prediction head for final classification, utilizing the previously learned feature representations. In this human-in-the-loop process, the model is iteratively trained on the most informative labeled data, with the algorithm selecting the most important samples for human annotation in each round. The figure (c) shows the importance score of the first iteration with entropy sampling. (d) This iterative cycle continues until satisfactory performance is achieved, resulting in final predictions of non-seizure and seizure annotation. This approach aims to achieve high classification performance with a significantly reduced number of annotations.

Our model consists of a ResNet50 backbone [33], a long short-term memory (LSTM) layer [34], and a multi-layer perceptron (MLP) for final classification (figure 2). Each component is trained in stages using using SSL and AL to minimize annotation needs while achieving high classification performance.

The ResNet backbone is trained using a SSL approach on the unlabeled data. This backbone extracts high-performance feature without relying on annotated data. The LSTM and MLP layers are then trained on a small pool of labeled samples. The LSTM layer learns the temporal patterns underlying each recording. The MLP serves as the classification head, predicting the corresponding labels, 2 classes for the RNS data and 3 classes for the NeuroVista data. We also implemented deep AL to identity the most informative samples, through query strategies. This layer ensures that only the most valuable data points are used to train the model, thereby reducing the need for extensive manual annotation. The hyperparameters and architectural details are provided in appendix B, while the data usage for both datasets is visualized in appendix C.

#### 2.3.1. Pre-processing data recordings

Minimal pre-processing was applied to facilitate clinical translation. Raw EEG data was normalized to the 0–1 range by scaling the voltage values for each subject across all recordings. Normalized data was split into 1 s sliding windows. Each window was resized to a 3 × 256 × 256 grayscale image. Data augmentation strategies were applied as described in appendix D.

#### 2.3.2. SSL for feature extraction

The ResNet50 backbone implements Swapping Assignments between Views (SwAVs), a contrastive SSL method [35]. The ResNet backbone was chosen as an off-the-shelf model and can be readily interchangeable with other architectures. SwAV is a contrastive SSL approach that, at a high level, expects larger and smaller crops from the same image to convey similar meanings. SwAV employs a ‘swapped’ prediction mechanism, where it predicts the cluster centroid of one view based on the representation of another view. This method enhances scalability and efficiency when handling large datasets, while also regularizing and enforcing consistency between cluster assignments for better clustering performance. To validate feature quality, the trained backbone is frozen, and a linear classifier is trained using labeled data for downstream tasks [36]. The backbone trained with SSL will be compared to a supervised learning-trained counterpart, which serves as a baseline benchmark for feature extraction performance. The comparison is based on classification accuracy for seizure detection using 1 s sliding windows.

#### 2.3.3. AL to reduce annotation effort

The study implements some of the most well-established deep AL algorithms. These strategies are grouped into four primary categories: uncertainty-based methods, which select high-uncertainty samples; representative-based methods, which select diverse samples; hybrid methods that combine elements of both; and a random selection baseline. The implementations is refactored using AL toolbox DeepAL+ [27]. The AL strategies are shown in table 1.

**Table 1.** Summary of active learning query strategies implemented in this study. The strategies are grouped into four categories: uncertainty-based methods, representative-based methods, hybrid approaches, and a random selection baseline. All methods were implemented using the DeepAL+ toolbox [27].

Strategy type	Strategies
Uncertainty-based method	Entropy (w/ Dropout)
	Margin (w/ Dropout)
	LeastConfidence (w/ Dropout)
	BALD
	BADGE
	LPL
Representative-based method	K-means, Coresets
Hybrid method	WAAL
Baseline benchmark	Random

We simulate the AL process retrospectively. The dataset was split 80–20 for training and validation, with a separate holdout consisting of 4 unseen patients as a testing set. Initially, 1% of the unlabeled training data was randomly selected and labeled. Subsequent rounds added 2% of the most informative samples, as determined by query strategies, to train the LSTM and MLP layers. The experiment was repeated for different query strategies. Data usage during the AL is visualized in appendix C.

AL methods were compared against two baselines to demonstrate their effectiveness and the benefits of SSL and AL. The first baseline, ‘Random Sampling with No Pre-training,’ involved randomly selecting samples for annotation and finetuning the model from ImageNet weights (utilizing PyTorch’s IMAGENET1K_V2 [37, 38]), simulating transfer learning with random sampling. The second, ‘Random Sampling with SSL Pre-training,’ also used random sample selection but incorporated pre-trained weights through SSL. These baselines helped to compare the advantages of AL strategies and SSL in boosting model performance and reducing the annotation burden.

We ensured consistency by initializing the LSTM and MLP layers with a fixed seed and saving the initial weights before training began. At each iteration, the checkpoint with the highest training accuracy guided the selection of new samples for labeling in the next round. After completing all rounds, we used the remaining unlabeled training data and the holdout dataset for final testing, evaluating accuracy on both the remaining unselected samples and the unseen patient samples.

To evaluate the effectiveness of each strategy, we analyzed the relationship between classification performance and the number of annotations, identifying strategies that achieved the highest accuracy with the fewest labels. Performance was tracked using the $F1$ score and area under the ROC curve (AUC), both of which treat expert annotations as the reference standard. These metrics allowed direct comparison across AL strategies and random sampling baselines. To ensure robustness, we validated the best-performing strategy on a holdout test set of unseen patient samples and further assessed its generalizability by applying it to the external NeuroVista dataset.

In addition to predictive accuracy, we also computed Cohen’s *κ* on a small separate subset of clips with multiple expert annotations, comparing algorithm—human agreement to human—human interrater reliability. While $F1$ and AUC are reported for the full dataset to reflect overall predictive accuracy, *κ* offers a complementary view of agreement under the assumption that human labels may be fallible. Because these metrics answer different questions, accuracy versus concordance, we report each in context to provide a more nuanced understanding of model performance.

#### 2.3.4. Combining queried samples with Kadane’s algorithm

In the AL process, each 1 s sliding window is evaluated using query strategy metrics, but labeling individual windows is impractical. Instead, we group and annotate continuous, information-rich regions using Kadane’s algorithm, which identifies subsets with the largest sum of metric values. Metrics from different strategies are normalized into an ‘Importance Score’ to ensure consistent application across strategies, as shown in figure 4(c). This approach dynamically selects high-value regions, optimizing the annotation process by prioritizing the most informative data for human review. For validation on the public NeuroVista dataset, Kadane’s algorithm was not applicable due to the discontinuous nature of the data, and instead, individual high-importance samples were directly selected for annotation.

## 3. Results

We analyzed a total of 84 720 iEEG recordings spanning 1176 h from two datasets. with over 11 000 annotations across two datasets with iEEG from two separate devices. The first dataset from the RNS system provided a robust foundation to test SSL and AL strategies. The second dataset, NeuroVista, served as an external benchmark to evaluate generalizability. On both these datasets, we rigorously tested our pipeline’s ability to reduce annotation requirements while maintaining high accuracy in detecting seizures.

### 3.1. SSL surpasses supervised performance

The SSL model outperformed the fully supervised baseline model on the RNS dataset in classifying seizure epochs. SSL model achieved an $F1$ Score of 0.9209 ± 0.019 compared to 0.8786 ± 0.025 for the supervised approach measured on 1 s window seizure detection classification to indicate that SSL may extract more pertinent features from unlabeled data.

**Figure 3.**
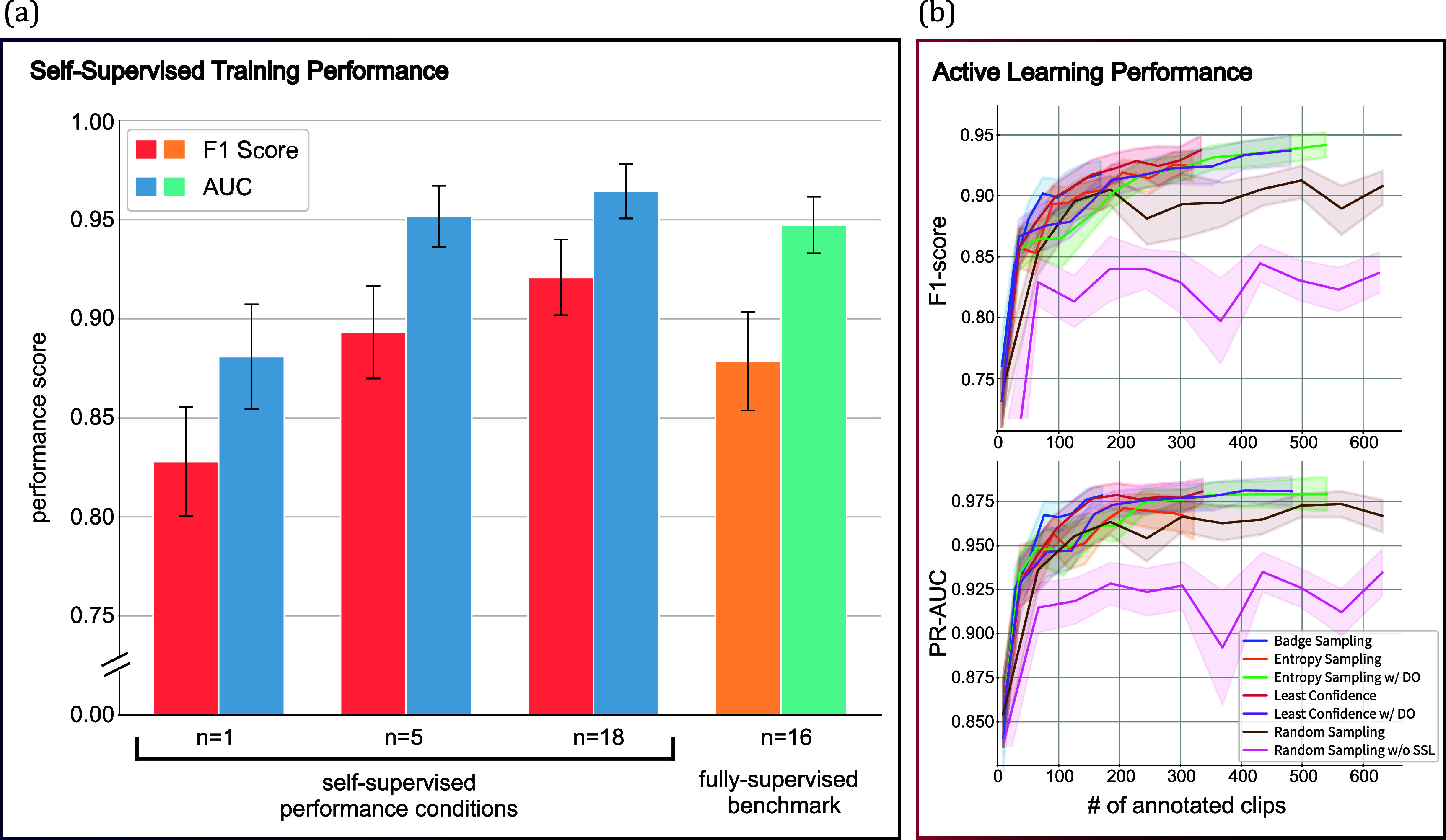
Performance evaluation of SSL and active learning strategies in iEEG classification (a) shows the linear classification performance of ResNet-50 model backbones trained under various self-supervised learning (SSL) conditions for 1 s EEG sliding window, in comparison to a fully-supervised benchmark. Initially, the ResNet-50 models were trained using SSL methods to learn feature representations from unlabeled data. Subsequently, the model weights were frozen, and a linear classifier was trained on labeled data to classify ictal and interictal states from 1 s iEEG window. For the fully-supervised benchmark, a ResNet-50 model pretrained on ImageNet (utilizing PyTorch’s IMAGENET1K_V2 [37, 38]) with unfrozen weights was directly fine-tuned on the labeled data. (b) shows the classification performance of the full pipeline using different active learning query strategies trained on different numbers of labeled segments. A ResNet-50 model, pretrained on 28 patients with SSL and an LSTM prediction head was used to perform the same task as in the (a). The benchmark of ‘Random Sampling’ simulates randomly annotating iEEG clips using SSL-pretrained weights, while ‘Random Sampling without SSL (Transfer Learning)’ represents randomly annotating samples with ImageNet-pretrained weights. Only the best performing strategies are shown, for the full list of performances see appendix F.

Performance ($F1$ Score and AUC) improved when we trained the SSL model by incrementally increasing the number of patients (*n* = 1 to *n* = 18). This indicates that exposure to diverse, unlabeled data allows the model to develop richer and more generalized feature representations (figure 3(a)). The SSL backbone also reduced the need for extensive retraining, as a simple classification could use the extracted features for downstream classification tasks. This efficiency highlights SSL’s potential to mitigate the need for large-scale labeled datasets, which are often challenging to obtain.

### 3.2. AL improves performance with fewer seizure annotations

AL reduced the number of annotations needed while maintaining high performance. The best-performing query strategy achieved an $F1$ Score of 0.947 ± 0.012, measure on classification accuracy of each 1 s sliding window, using just 336 annotations. In comparison, random sampling baselines required significantly more annotations and achieved lower $F1$ Scores, ranging from 0.89 to 0.91 with SSL pretraining and 0.82–0.84 with transfer learning. These results demonstrate the efficiency of AL in selectively annotating the most informative samples (figure 3(b)).

The model’s performance also exceeded human benchmarks in inter-rater reliability. The optimal AL strategy achieved a Cohen’s Kappa of 0.9510 ± 0.0429, surpassing the human annotators’ reliability of 0.9263 ± 0.0434 for detecting ictal events in 90 s recordings (details in appendix H). This suggests that AL can yield a model whose consistency rivals or even exceeds that of human experts, reducing the variability often introduced by subjective interpretations. This measure is used because unlike the $F1$ Score, which assumes human annotations are the ground truth, Cohen’s Kappa accounts for inter-rater reliability, effectively addressing annotation noise and bias.

Among the tested strategies, uncertainty-based methods, which prioritize high-uncertainty examples for annotation, consistently outperformed representative-based approaches. This emphasizes the importance of targeted sampling in optimizing model training efficiency. Additionally, the higher performance of SSL-pretrained baselines compared to transfer-learned baselines highlights SSL’s effectiveness in generating information-rich embeddings, further improving the AL process.

### 3.3. Embedding visualization and prediction analysis

**Figure 4.**
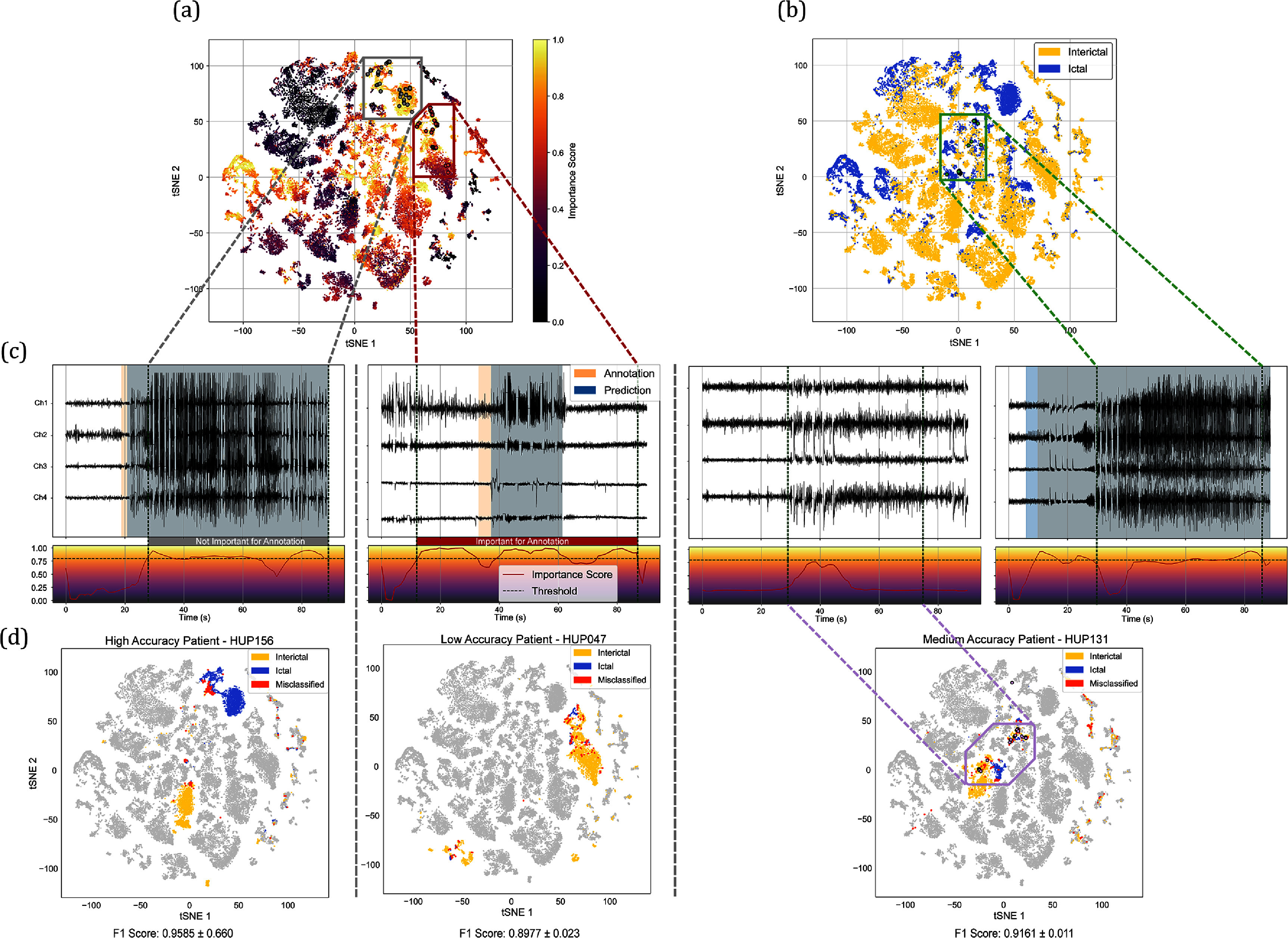
Active learning query visualization and prediction analysis (a) feature representations of 1 s iEEG windows are shown with a color-coded scale indicating the importance scores of unlabeled samples. In the active learning framework, samples with the highest importance scores are prioritized for human annotation during each training iteration. Different active learning strategies generate varying importance scores, which are updated in successive training rounds. (b) Ground truth labels of 1 s iEEG windows annotated by a human expert. (c) Ground truth and predicted annotations for example RNS episodes from three patients are shown, visualizing the algorithm’s process for selecting important regions for annotation. Kadane’s algorithm aggregates the importance scores, identifying and prioritizing the continuous regions with the highest overall importance for annotation. More example of classification can be found in appendix G (d) per-patient feature representation distributions are depicted. Examples from three patients with different prediction accuracies are shown to visualize the embedding of misclassified data.

The feature embeddings generated by the SSL backbone on 1 s sliding window revealed clear separations between ictal and interictal samples, as shown in figure 4. Episodes with distinct cluster separations in the embedding space corresponded to higher classification accuracy, while less distinct separations were associated with lower accuracy. This indicated that SSL embeddings are robust in capturing the underlying structure of data.

Figure 4(c) shows examples of correctly classified and misclassified samples to illustrate the model’s ability to handle diverse signal characteristics. Patients with varying classification performance (low, medium, and high) demonstrated that well-separated embeddings align with better predictions, as summarized in table 2.

**Table 2.** Patient information and per-patient classification performance. The table presents patient information and per-patient prediction performance, as well as the corresponding annotations used by the active learning algorithm. The last four patients in the table are included to demonstrate the classification performance on previously unseen patients.

ID	Sex	Age at onset	Laterality	Lead locations	Age at RNS implant	sz Foci	# Annotated clips	# Used clips	F1 score	AUC
HUP047	M	17	R	N	54	frontal	126	22	$0.8977\pm0.023$	$0.9746\pm0.015$
HUP084	M	1	B	M	55	L. Hipp/ R. Hipp	70	24	$0.9115\pm0.015$	$0.9652\pm0.015$
HUP096	F	35	B	N	50	temporal	160	13	$0.9511\pm0.010$	$0.9791\pm0.012$
HUP109	M	42	B	M	61	L. Hipp/ R. Hipp	186	24	$0.9185\pm0.013$	$0.9700\pm0.012$
HUP121*	F	5	L	N	52	parietal/frontal	103	12	$0.7132\pm0.031$	—
HUP129	M	30	R	B	41	R. Hipp/ R Insula	182	4	$0.9346\pm0.012$	$0.9686\pm0.012$
HUP131	M	3	L	B	29	frontal	132	23	$0.9161\pm0.011$	$0.9440\pm0.026$
HUP137	M	36	B	M	53	L. Hipp/ R. Hipp	144	43	$0.9450\pm0.008$	$0.9844\pm0.009$
HUP147	F	12	L	N	45	parietal/insula	259	32	$0.9196\pm0.016$	$0.9891\pm0.006$
HUP156	F	12	L	N	44	temporal	173	9	$0.9585\pm0.660$	$0.9979\pm0.001$
HUP159	M	17	L	N	23	temporal	251	89	$0.9043\pm0.006$	$0.9715\pm0.008$
HUP182	F	19	B	B	26	L Het. / R. Hipp	166	16	$0.9158\pm0.017$	$0.9741\pm0.016$

HUP197 ^*^	F	1	L	N	41	Temporal	54	0	$0.8998\pm0.200$	—
HUP199	F	8	B	B	45	L. Hipp/ R. Hipp	90	0	$0.9100\pm0.009$	$0.9694\pm0.006$
RNS026	M	21	B	M	24	L. Hipp/ R. Hipp	262	0	$0.9241\pm0.007$	$0.9726\pm0.013$
RNS029	F	23	B	M	46	L. Hipp/ R. Hipp	151	0	$0.9401\pm0.008$	$0.9785\pm0.007$

* means the patient only has one class of annotations.

### 3.4. Robust performance on unseen patients

Our pipeline generalized to unseen patients and demonstrated classification performance comparable to that of previously seen patients. In the test set, certain patients were deliberately excluded from both the pre-training and AL phases to simulate real-world scenarios where models are applied to new patients. Despite these exclusions, the model maintained robust classification accuracy, highlighting its adaptability and potential for deployment in diverse clinical contexts (table 2).

### 3.5. Generalization to external datasets

To assess generalizability, the pipeline was externally validated on the NeuroVista dataset—a public dataset for seizure detection. The model achieved a mean AUC of $0.9628 \pm 0.015$ calculated as the average of the early onset—interictal and ictal—interictal AUCs, which is comparable to the competition-winning algorithm’s AUC of 0.9667. Notably, this performance was achieved while using only one-seventh of the labeled data (1050 samples versus 7600), underscoring the effectiveness of our AL strategy in significantly reducing annotation requirements.

While the NeuroVista dataset’s discontinuous nature prevented the application of techniques of Kadane’s algorithm, the pipeline adapted effectively by selecting and prioritizing the most informative individual samples during AL. These results reinforce the pipeline’s ability to generalize to external datasets without extensive fine-tuning while substantially reducing annotation requirements. The detailed result can be found in appendix I.

## 4. Discussion

Annotating large data sets from implantable or wearable devices is a daunting task that is essential for diverse purposes: comprehensive annotations are required for FDA device and algorithm approvals, clinician annotations guide patient care, and high-quality annotations support research analysis. We present a new HITL approach to streamline the annotation process, applied as a proof-of-concept to classify seizure recordings from two implantable brain devices. iEEG recordings involve complex dynamics, large data volumes, and high annotation costs. While machine learning methods exist, they often require substantial labeled data to be effective. Our approach reduces this burden by having human experts iteratively annotate only the most informative data segments, optimizing training and significantly reducing workload. Minimal preprocessing is applied to raw EEG data, and a self-supervised deep learning model eliminates the need for manual labeling and feature extraction while achieving high-quality embeddings. Deep AL refines this process by repeatedly querying only the most informative clips, updating the model after each round, and steadily boosting performance. As a result, the pipeline reaches human-level accuracy while using only a small fraction of the labeled data. On the RNS dataset, for instance, Sharanya *et al* [15] trained a 2D-CNN on ${\sim}138\,000$ fully annotated episodes for binary seizure classification and then fine-tuned on ${\sim}1000$ episodes to localize onset times. In contrast, our self-supervised pretraining eliminates the need for episode-level labels altogether; we fine-tune only on the actively selected segments, further reducing annotation effort. The HITL training scheme is designed so that other detector architectures can reap the same benefits. Other model architectures, such as *ChronoNet* [39], *SPaRCNet* [14], as well as transformer-based designs such as *EEG-Former* [40] can be incorporated with only minor changes to our provided training scaffold. Evaluations on the publicly available NeuroVista benchmark confirm the pipeline’s ability to generalize beyond the RNS data. All code is released at this GitHub repository.

### 4.1. Clinical applications

The proposed HITL workflow reduces the burden of annotation by algorithmically processing each continuous recording. Only segments identified by the detector as ambiguous or atypical are forwarded to a neurologist or epileptologist for review, allowing routine background patterns to be handled automatically. This strategy has reduced the number of clips requiring expert scrutiny by approximately six-fold when training a model from scratch. Once deployed, the pipeline can yield even greater efficiency, accelerating clinical workflows without compromising diagnostic accuracy.

By surfacing only clinically salient or uncertain events, the system enables specialists to focus on complex seizures, treatment adjustments, and surgical candidacy evaluations rather than manually scanning entire recordings. Corrections made during routine review are integrated into incremental model updates, allowing rapid adaptation to patient-specific signal morphologies that often confound general-purpose detectors. This capability is especially valuable for patients with evolving seizure patterns or atypical electrode configurations. Because the pipeline can be calibrated with sparsely labeled data, new centers adopting the model do not need to retrain from scratch, a small site-specific calibration set is sufficient. This facilitates deployment across hospitals with diverse patient populations and recording setups.

The triage loop remains active after deployment, ingesting new recordings and continuously refining decision boundaries. As the proportion of flagged segments decreases over time, the workflow moves toward autonomous monitoring while retaining a clear path for human intervention when novel patterns emerge or research questions arise. Thus, HITL offers an immediate reduction in annotation workload, supports patient-specific tuning, and serves as a pragmatic bridge toward fully autonomous seizure-tracking systems that maintain clinical oversight.

### 4.2. Broad application to other domains

As the number of neuro and other medical devices on the market that generate digital data continues to grow, some even capable of ultra-long-term continuous recording [41], there is a pressing demand for annotation strategies that scale with the data they generate. Our HITL pipeline addresses this challenge by streamlining annotation and can aid the development of detection algorithms for neural events. Although demonstrated here for ictal events, the same workflow can be generalized seamlessly to other clinically relevant biomarkers. For instance, in epilepsy research, it can accelerate the detection of high-frequency oscillations [42, 43] and interictal epileptiform discharges (‘spike detection’) [44, 45]. In critical-care and intra-operative monitoring, it can sift through continuous neurophysiology recordings to surface emergent patterns that demand immediate attention [46, 47]. Crucially, as multimodal devices proliferate and datasets combine EEG, hemodynamics, motion, and wearable metrics [48, 49], manual labeling becomes an even greater bottleneck. The efficiency and adaptability of our HITL pipeline enable researchers and clinicians to incorporate novel biosignals without incurring prohibitive annotation costs, thereby speeding biomarker discovery, device algorithm development, and translation into routine care.

### 4.3. Limitations and future work

Our approach has several limitations that need addressing. First, despite standardized efforts, inter-rater reliability remains a challenge, with different raters providing varying annotations that we accept as ground truth, introducing noise and bias into the data [8]. Improving accuracy could involve a judicious choice of experts or a group consensus for annotations.

Furthermore, annotations obtained prior to the experiment restrict our AL process to a pre-annotated subset of the dataset, limiting data exploration and potentially affecting the overall accuracy. Prospective studies that integrate our HITL approach could better showcase the potential for enhanced accuracy and reduced annotation.

Additionally, although we have addressed the class imbalance common in seizure recordings, the effectiveness of our pipeline in real-world applications where interictal events typically outnumber ictal events remains uncertain [50]. Our current models also struggle with covariate shift across different datasets without specific tuning. Although the pipeline currently uses a 2D ResNet CNN as its backbone, this component is interchangeable; alternative architectures such as time series transformers could potentially deliver even stronger performance. [51–54].

While AL boosts annotation efficiency by targeting uncertain and diverse samples, it can overlook examples where the model is overly confident yet wrong, creating blind spots in prediction accuracy. To address this, future work could use conformal prediction frameworks to provide theoretical guarantees for rigorous error control and calibrated confidence sets [55], enabling AL process to flag overconfident predictions for manual review and close these performance blind spots.

To enhance accessibility in clinical settings, we plan to develop a web application for uploading and annotating EEG recordings, focusing on safety, transparency, and explainability [55–58]. This will allow clinicians and researchers to use the tool confidently in real-world workflows.

## 5. Conclusion

Our work highlights the potential of integrating human expertise with advanced machine learning to address challenges in annotating large-scale neurophysiological data to pave the way for broader applications in clinical and research neurotechnology. The era of machine learning is poised to greatly accelerate the development of algorithms and devices for research and medical applications, particularly those generating large data streams requiring expert annotation and validation. We believe the above human in the loop strategy holds great promise for these tasks. It also provides another opportunity to reconsider what constitutes a human ‘expert,’ and the role of experts in benchmarking algorithms, devices and their applications. It is possible that these techniques, and their rigor, may further limit or even replace humans in applications where rigorous review is required.

## Figures and Tables

**Figure 5.**
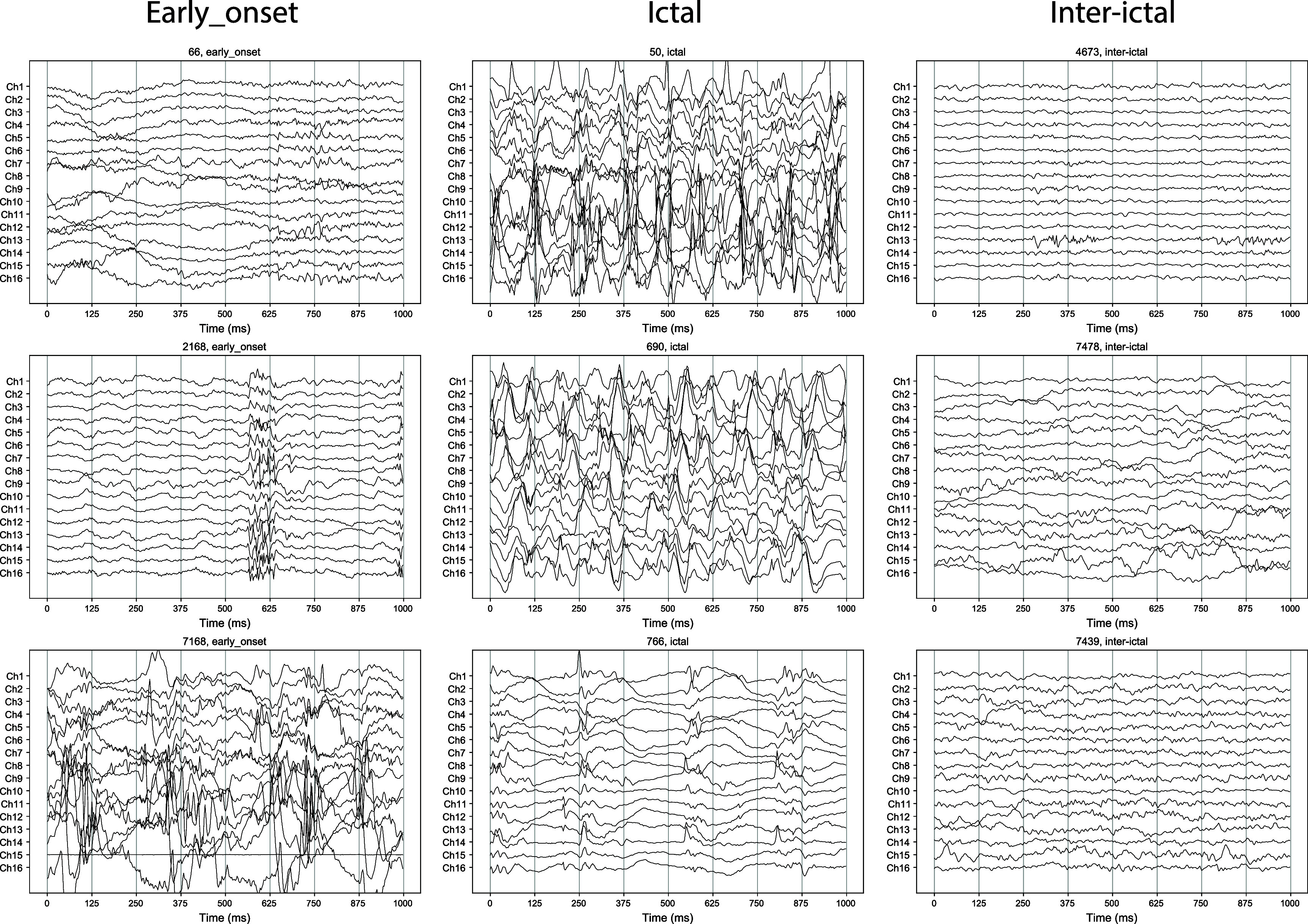
A sample of data randomly selected from the NeuroVista dataset. The original NeuroVista data has been reformatted into discrete 1 s sliding windows in the competition. The above panel represents a 1 s sliding window along with corresponding class labels.

**Figure 6.**
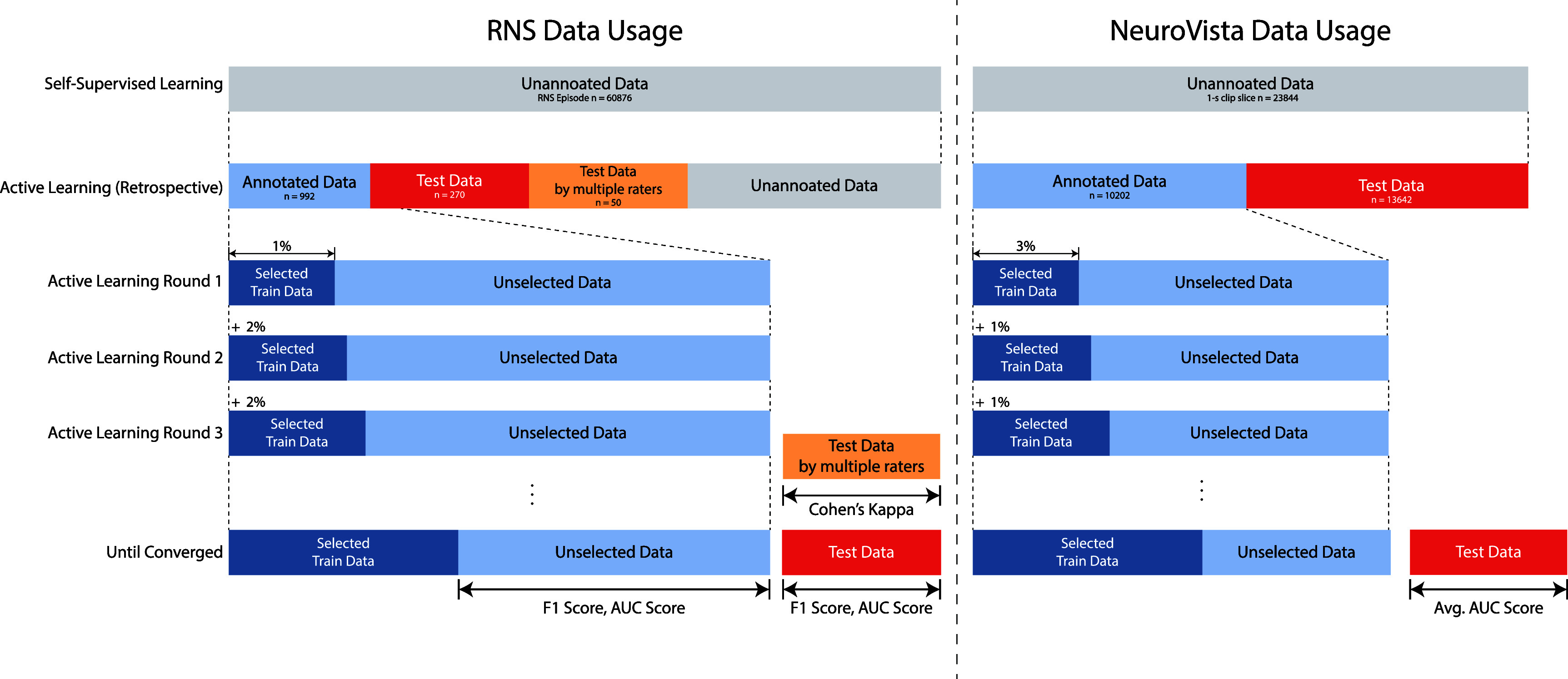
Data usage of RNS and NeuroVista datasets in this study. For the RNS dataset, unannotated episodes were used for unsupervised pretraining. Then, 80% of the annotated data was used for the active learning training process, and 20% was held out as a test set. A separate test set, annotated by multiple raters, was used for inter-rater agreement analysis. During the retrospective active learning process, new samples were iteratively introduced until convergence. $F1$ score and AUC are reported for the test data and the unselected training data, while Cohen’s *κ* is reported for the test set annotated by multiple raters. For the NeuroVista dataset, the entire dataset was used for self-supervised training. Annotated and test splits were defined by the dataset publisher, and performance was evaluated using the metrics specified by the publisher.

**Figure 7.**
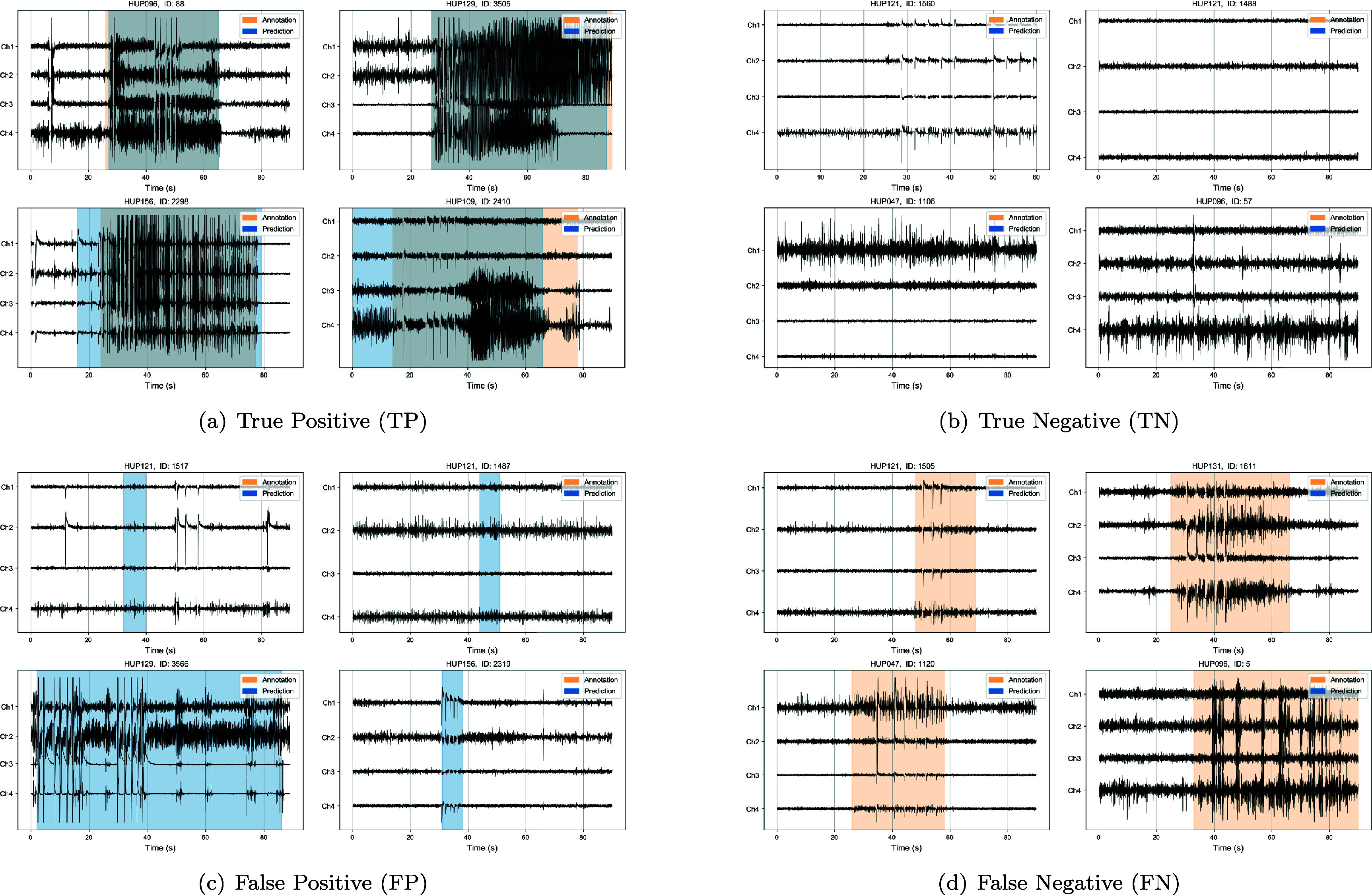
Randomly selected prediction examples from the RNS dataset. The upper panel presents four types of prediction outcomes—True Positive (TP), True Negative (TN), False Positive (FP), and False Negative (FN)—which have been randomly selected for demonstration.

**Figure 8.**
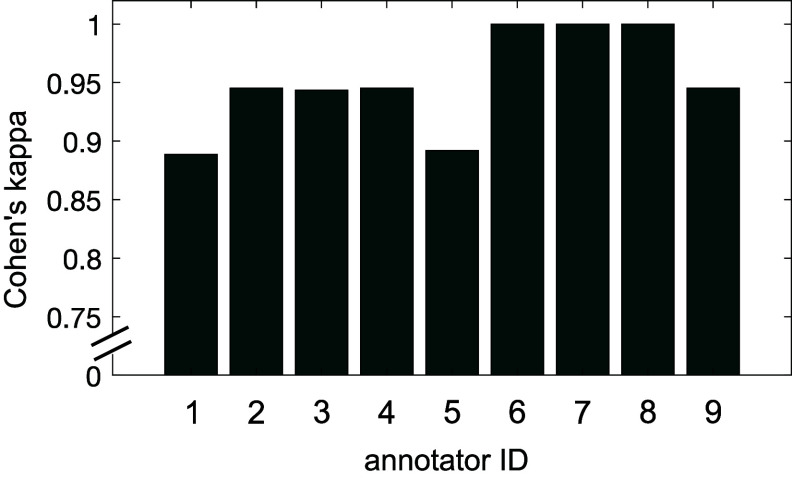
Individual machine-annotator performance on a test set of EEG clips from three patients. The average Cohen’s kappa agreement between machine predictions and human annotations was 0.95 ± 0.04.

**Figure 9.**
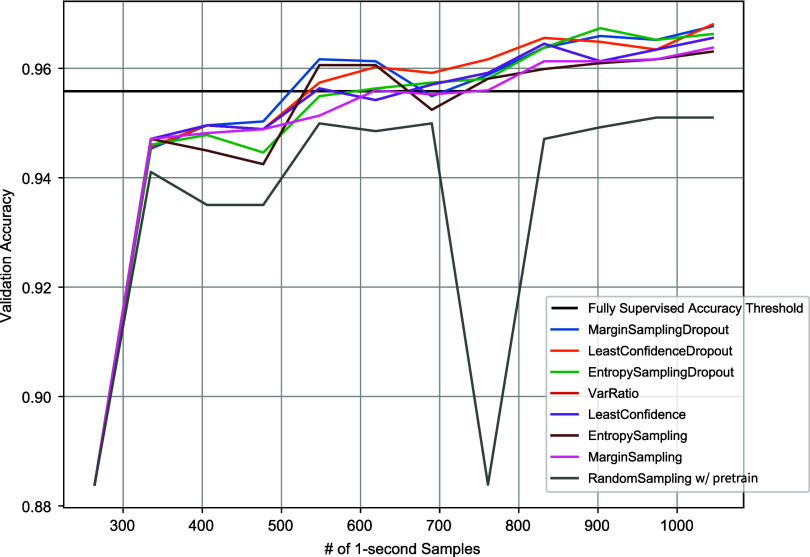
Classification accuracy on the validation set for the best-performing query strategies applied to the NeuroVista dataset. Similar to the results observed with the RNS dataset, these strategies demonstrate a clear improvement over the random sampling baseline.

## Data Availability

The code supporting these findings is available at the following GitHub repository: https://github.com/penn-cnt/RNS_Annotation-Pipeline. The NeuroVista data utilized in this study can be accessed on the Kaggle competition page: www.kaggle.com/competitions/seizure-detection. The RNS data used in this study will be available upon request, subject to Institutional Review Board approval. All data that support the findings of this study are included within the article (and any supplementary files).

## Appendix B Training configuration

### B.1. SSL with SwAV

#### B.1.1. Model Architecture

The model used in this study is a deep neural network composed of the following layers:

• Input dimension: $3\times256\times256$
• Model backbone: ResNet50 without projection layer
• Projection head:
  – Layer 1: Linear Layer (2048, 2048), BatchNorm, ReLU
  – Layer 2: Linear Layer (2048, 128)
• Prototype:
  – Number of prototypes: 2048
  – Length of each prototype: 128
  – Unfrozen prototype at epoch: 1
• Negative memory bank:
  – Size of memory bank: 512
  – Introduce memory bank at epoch: 80

#### B.1.2. Training hyperparameters

The model was trained with the following hyperparameters:

• Optimizer: Adam
• Learning rate: 0.001
• Batch size: 340
• Epochs: 150
• Loss function: SwAV Loss
  – Sinkhorn *ε*: 0.05
• Learning rate schedule: cosine decay

### B.2. Active learning

#### B.2.1. Model architecture

The model used in this study is a deep neural network composed of the following layers:

• Input dimension: $\texttt{sequence}\_\texttt{length}\times3\times256\times256$
• Model backbone: **Frozen** ResNet50 without projection layer
• LSTM and projection head:
  – Layer 1: Linear Layer (2048, 256), ReLU
  – Layer 2: Bidirectional LSTM (input size = 256, hidden size = 128)
  – Layer 3: Linear Layer(256, 64), ReLU
  – Classification Layer: Linear Layer(64, # of classes)

#### B.2.2. Training hyperparameters

The model was trained with the following hyperparameters:

• Optimizer: Adam
• Learning rate: 0.001
• Batch size: 8
• Epochs: 60
• Loss function: Focal Loss
  – *α*: 1
  – *γ*: 5

### B.3. Training hardware

Model training and evaluation were performed on the following hardware setup:

#### B.3.1. System 1

• GPU: NVIDIA RTX 4090, 24 GB
• CPU: AMD Ryzen 7 5800X 8-Core Processor
• RAM: 128 GB
• Operating system: Windows 10
• Framework: PyTorch 2.6.0, CUDA 12.6

#### B.3.2. System 2

• GPU: NVIDIA A40, 48 GB
• CPU: AMD EPYC 7502P 32-Core Processor
• RAM: 256 GB
• Operating system: openSUSE Leap 15.6
• Framework: PyTorch 2.6.0, CUDA 12.6

### B.4. Reproducibility control

To ensure reproducibility of the experiments, all sources of randomness were controlled. Specifically, the random seed was fixed for Python’s built-in random module, NumPy, and PyTorch. The PyTorch DataLoader was also configured with a fixed seed through the worker_init_fn. Additionally, CUDA operations were set to deterministic mode and disabled benchmark mode.

## Appendix D Data augmentation strategies and parameters

Data Augmentation is used to increase the diversity of data available for training by generating new artificial data based on existing training data. Data augmentation helps the model to learn more general features and improve model robustness.

• Channel swapping
• Color jitter randomly apply, probability 0.5
• Gaussian blur random apply, probability 0.5
• Invert voltage, random apply, probability 0.2
• Posterize signal, random apply, probability 0.2

## Appendix E AL strategies

The study implements some of the most well-established deep AL algorithms, encompassing a wide range of query strategies based on distinct principles. These strategies are grouped into four primary categories: **Uncertainty-based methods**, **Representative-based methods**, **Hybrid methods**, and **Baseline benchmarks**. The implementations is refactored using AL toolbox DeepAL+ [27].

• **Uncertainty-based methods**: uncertainty-based methods are designed to prioritize data points where the model exhibits the highest uncertainty, aiming to maximize the model’s learning efficiency. Examples include:
  – **Entropy (with Dropout)** [59]: selects samples with the highest entropy to capture diverse and uncertain predictions.
  – **Margin (with Dropout)** [60]: focuses on the smallest difference between the two most probable predictions to identify ambiguous cases.
  – **Least confidence (with Dropout)** [61]: targets the samples with the lowest model confidence in its predictions.
  – **Bayesian AL by disagreement (BALD)** [62, 63]: utilizes Bayesian methods to select samples with maximum information gain.
  – **BADGE** [64]: combines uncertainty sampling and diversity by leveraging gradients for query selection.
  – **Loss prediction loss (LPL)** [65]: a method that aims to predict the model’s training loss for unlabeled data and selects samples with the highest predicted loss.
• **Representative-based methods**: representative-based methods aim to ensure that the selected samples are diverse and representative of the entire dataset. Techniques employed include:
  – **K-means** [66]: clustering method used to identify representative samples by grouping data points based on their feature similarity.
  – **Coresets** [67]: focuses on selecting a subset of data points that best approximate the entire dataset, preserving its global structure.
• **Hybrid methods**: hybrid methods combine the strengths of multiple principles, balancing both uncertainty and representativeness. For example:
  – **WAAL** [68]: integrates uncertainty estimation with adversarial learning to enhance the selection of informative and representative samples.
• **Baseline benchmark**: baseline benchmarks serve as a reference to evaluate the performance of the advanced strategies. The study includes **Random Sampling** as the baseline, which selects samples randomly without any prioritization.

## Appendix F RNS dataset AL full performance

**Table:**

Method	Metric	1	2	3	4	5	6	7	8	9	10	11
BadgeSampling	n_sample	9	29	53	76	100	122	145	171	—	—	—
	acc_std	0.7696 ± 0.023	0.8515 ± 0.015	0.8908 ± 0.014	0.9117 ± 0.013	0.9083 ± 0.015	0.9156 ± 0.016	0.9236 ± 0.012	0.9276 ± 0.011	—	—	—

BALDDropout	n_sample	9	87	150	225	254	300	384	454	518	587	671
	acc_std	0.7428 ± 0.023	0.8853 ± 0.014	0.4234 ± 0.006	0.8757 ± 0.023	0.8799 ± 0.013	0.4196 ± 0.006	0.8948 ± 0.014	0.8872 ± 0.02	0.8777 ± 0.023	0.9037 ± 0.014	0.4123 ± 0.005

EntropySampling	n_sample	9	37	64	92	117	143	175	207	250	290	322
	acc_std	0.7414 ± 0.022	0.8675 ± 0.017	0.8624 ± 0.017	0.9028 ± 0.015	0.9039 ± 0.013	0.912 ± 0.013	0.9146 ± 0.016	0.9289 ± 0.011	0.9242 ± 0.012	0.9354 ± 0.01	0.9343 ± 0.013

EntropySampling w/ DP	n_sample	9	36	64	107	163	194	240.0	307	355	429	540.0
	acc_std	0.7414 ± 0.022	0.8647 ± 0.017	0.8736 ± 0.014	0.875 ± 0.024	0.8972 ± 0.013	0.9133 ± 0.012	0.927 ± 0.012	0.9329 ± 0.011	0.9411 ± 0.01	0.9446 ± 0.011	0.9515 ± 0.01

KleftGreedyPCA	n_sample	9	36	74	140.0	205	259	300.0	352	406	455	510.0
	acc_std	0.7347 ± 0.023	0.7689 ± 0.051	0.8607 ± 0.027	0.8548 ± 0.017	0.8805 ± 0.027	0.4061 ± 0.007	0.9106 ± 0.014	0.881 ± 0.022	0.4 ± 0.007	0.9139 ± 0.012	0.8693 ± 0.032

KMeansSampling	n_sample	9	35	66	101	144	179	219	294	391	433	486
	acc_std	0.7335 ± 0.024	0.8705 ± 0.018	0.8794 ± 0.017	0.8691 ± 0.019	0.8856 ± 0.018	0.8539 ± 0.018	0.8909 ± 0.019	0.9075 ± 0.015	0.9065 ± 0.016	0.9168 ± 0.015	0.9129 ± 0.018

LeastConfidence	n_sample	9	38	65	96	125	156	196	230.0	266	301	336
	acc_std	0.7414 ± 0.022	0.8671 ± 0.015	0.8881 ± 0.014	0.9083 ± 0.011	0.9167 ± 0.013	0.9267 ± 0.012	0.9324 ± 0.012	0.9382 ± 0.01	0.9341 ± 0.016	0.9385 ± 0.011	0.9471 ± 0.012

LeastConfidence w/ DP	n_sample	9	37	82	121	158	189	236	289	354	406	483
	acc_std	0.7414 ± 0.022	0.8763 ± 0.014	0.8852 ± 0.02	0.8882 ± 0.013	0.9055 ± 0.013	0.9224 ± 0.013	0.9261 ± 0.011	0.9322 ± 0.013	0.9336 ± 0.015	0.9428 ± 0.012	0.9466 ± 0.013

LossPredictionLoss	n_sample	9	35	60.0	86	106	136	158	187	207	230.0	252
	acc_std	0.7147 ± 0.04	0.7459 ± 0.039	0.7672 ± 0.037	0.8007 ± 0.031	0.8198 ± 0.036	0.8128 ± 0.028	0.7694 ± 0.027	0.8186 ± 0.021	0.8387 ± 0.027	0.7774 ± 0.035	0.8331 ± 0.028

MarginSampling w/ DP	n_sample	9	48	82	133	158	186	219	264	311	378	440.0
	acc_std	0.7428 ± 0.023	0.8436 ± 0.014	0.9055 ± 0.013	0.8785 ± 0.024	0.8947 ± 0.031	0.9115 ± 0.012	0.9194 ± 0.013	0.929 ± 0.012	0.9377 ± 0.01	0.9398 ± 0.011	0.9442 ± 0.01

MeanSTD	n_sample	9	71	153	226	313	401	470.0	547	635	725	814
	acc_std	0.7576 ± 0.024	0.4148 ± 0.008	0.8976 ± 0.018	0.8864 ± 0.021	0.8997 ± 0.018	0.9078 ± 0.015	0.9042 ± 0.023	0.9091 ± 0.016	0.4138 ± 0.007	0.9265 ± 0.014	0.9276 ± 0.015

WAAL	n_sample	9	45	79	130.0	159	212	287	353	440.0	507	588
	acc_std	0.7179 ± 0.022	0.741 ± 0.049	0.7535 ± 0.029	0.8211 ± 0.027	0.8107 ± 0.028	0.81 ± 0.034	0.8352 ± 0.023	0.8079 ± 0.044	0.8327 ± 0.036	0.811 ± 0.037	0.7997 ± 0.03

RandomSampling	n_sample	9	66	126	185	245	303	369	435	499	565	632
	acc_std	0.7428 ± 0.023	0.8536 ± 0.017	0.8956 ± 0.015	0.9053 ± 0.012	0.8815 ± 0.02	0.8933 ± 0.027	0.8944 ± 0.018	0.9056 ± 0.012	0.9128 ± 0.013	0.8896 ± 0.02	0.9081 ± 0.014

RandomSampling w/o SSL	n_sample	9	66	126	185	245	303	369	435	499	565	632
	acc_std	0.5725 ± 0.106	0.8266 ± 0.022	0.8088 ± 0.024	0.8388 ± 0.03	0.8387 ± 0.018	0.8263 ± 0.028	0.7906 ± 0.04	0.844 ± 0.017	0.8286 ± 0.019	0.8198 ± 0.02	0.8352 ± 0.019
